# Automated Quantification of Head Motion Synchrony in Peer Interactions of Autistic and Non‐Autistic Youth

**DOI:** 10.1111/desc.70285

**Published:** 2026-09-14

**Authors:** Kathryn A. McNaughton, Heather A. Yarger, Elizabeth Redcay

**Affiliations:** ^1^ Neuroscience and Cognitive Science Program University of Maryland College Park College Park USA; ^2^ Department of Psychology University of Maryland College Park, USA; ^3^ Department of Psychology James Madison University College Park, Harrisonburg USA

**Keywords:** adolescence, autism, computer vision, head motion, peer interaction, synchrony

## Abstract

Peer interactions take on increasing importance for youth in middle childhood and adolescence. Autistic youth face particular challenges interacting with non‐autistic peers; thus, it is critical to understand factors that predict interaction enjoyment for autistic and non‐autistic youth. One candidate predictor is interpersonal motion synchrony. Experimentally induced motion synchrony leads to interpersonal liking and closeness, but these outcomes from experimental synchrony may not generalize to synchrony that naturally occurs in interpersonal interactions. Computer vision approaches allow for non‐invasive quantification of synchrony in developmental populations, and thus provide an opportunity to investigate the role of naturalistic motion synchrony in peer interactions between autistic and non‐autistic youth. Autistic and non‐autistic youth were paired into three dyad types (autistic with autistic, autistic with non‐autistic, and non‐autistic with non‐autistic) and completed a brief video‐recorded “getting‐to‐know‐you” interaction. Head position was extracted frame‐by‐frame from video recordings using OpenPose. Cross‐wavelet coherence was computed to quantify head motion synchrony. We found significant head motion synchrony in interacting (compared to time‐scrambled) dyads, but levels of synchrony did not differ by dyad type. Across dyad types, we found evidence that head motion synchrony negatively predicts youth‐reported enjoyment and positively predicts youth‐reported discomfort in the interaction. Unlike prior findings of relations between synchrony and positive outcomes, here, synchrony predicted negative conversational outcomes. Thus, these findings add nuance to our understanding of the role of synchrony in naturalistic interactions. They also demonstrate the application of non‐invasively quantifying synchrony in minimally structured peer interactions including autistic youth using computer vision.

## Summary

- Computer vision can be used to detect naturally occurring head motion synchrony in peer dyads of interacting youth.
- Head motion synchrony does not differ across peer dyad types (autistic youth paired with autistic youth, autistic paired with non‐autistic, non‐autistic paired with non‐autistic).
- Increased head motion synchrony predicts increased youth‐reported discomfort and decreased youth‐reported enjoyment of the interaction.

## 1 Introduction

Navigating peer interactions is an important aspect of youth well‐being as peer interactions take on increasing importance throughout middle childhood and adolescence (Alsarrani et al. 2022; Mitic et al. 2021). Autistic youth experience challenges in peer interactions that impact their mental health and well‐being (O'Connor et al. 2022; Storch et al. 2012). Therefore, understanding factors that impact peer interaction enjoyment for autistic youth is particularly important. Synchronizing movements with others is a fundamental aspect of social interactions and impacts the outcomes of the social interaction (Shamay‐Tsoory et al. 2019). Thus, examining interpersonal motion synchrony between interaction partners provides the opportunity to shed light on interaction challenges between autistic and non‐autistic individuals. Computational approaches offer opportunities for non‐invasively and efficiently quantifying synchrony, providing the potential to identify the ways in which synchrony impacts peer interactions for autistic and non‐autistic youth, yet no studies have yet used these approaches within autistic adolescent peer interactions.

Interpersonal motion synchrony, the collection of time‐ and form‐ aligned movements that occur in social interactions, is a core component of social interactions (Louwerse et al. 2012; Shamay‐Tsoory et al. 2019). Substantial research has demonstrated relations between experimental induction of synchrony (e.g. finger tapping in synchrony with a partner) and positive interpersonal outcomes such as social‐cognitive task performance, positive affect, and prosocial behavior across the lifespan (Cirelli 2018; Mogan et al. 2017; Rennung and Göritz 2016; Vicaria and Dickens 2016), including demonstrating relations in youth peer interactions between experimentally induced synchrony and positive interpersonal outcomes (Rabinowitch and Knafo‐Noam 2015; Tunçgenç and Cohen 2016). While these relationships between experimentally induced synchrony and social outcomes have been established, they may not generalize to the synchrony that naturally occurs in interpersonal interactions. In typical conversation settings, partners synchronize their body movements (Ayache et al. 2021; Louwerse et al. 2012), including their head motion (Hadley and Ward 2021; Hale et al. 2020) and overall degree of body motion (Fujiwara and Daibo 2016; Georgescu et al. 2020). Yet studies directly linking naturally occurring motion synchrony to positive interpersonal outcomes have yielded mixed results. Some previous work has linked increased whole‐body synchrony in conversations between unfamiliar adults and positive interaction outcomes like rapport and positive affect (Fujiwara et al. 2020; Tschacher et al. 2014), while other work has found no relations (Georgescu et al. 2020). Additional research is needed to understand interaction outcomes associated with naturally evoked synchrony.

Interpersonal motion synchrony may contribute to interaction challenges between autistic and non‐autistic individuals. Motion synchrony is reduced in social interactions between autistic and non‐autistic individuals compared to interactions between non‐autistic individuals across a variety of contexts and tasks (Carnevali et al. 2024; Glass and Yuill 2023; McNaughton and Redcay 2020). Nevertheless, there has been minimal work linking naturally occurring synchrony in these mismatched (autistic/non‐autistic) dyads and interaction outcomes. Some work demonstrates a positive effect of synchrony on either interaction enjoyment or positive impressions of the interaction. For example, smiling synchrony predicts increased youth‐reported interaction enjoyment across dyads of autistic and neurotypical youth (McNaughton et al. 2024), and increased head motion synchrony in which one partner “led” the other partner contributes to better third‐party impression ratings of non‐autistic interaction partners in interactions between autistic and non‐autistic adults (Plank et al. 2023). However, other work demonstrates no effect of synchrony on conversation outcomes. For example, whole‐body synchrony in conversations between autistic and neurotypical adults did not relate to participant‐rated pleasantness of the interaction (Georgescu et al. 2020). In another study, whole‐body synchrony did not predict participant‐rated rapport in matched non‐autistic dyads, mismatched non‐autistic/autistic dyads, or matched autistic dyads (Efthimiou et al. 2025). However, there were differences between autistic and non‐autistic dyads on the strength of the non‐significant relations between synchrony and rapport (Efthimiou et al. 2025), highlighting the need to further characterize relations between synchrony and interaction outcomes in interactions involving autistic individuals.

Head motion synchrony may be particularly relevant to understanding interaction outcomes between autistic and non‐autistic individuals. Head motion conveys important social signals (Duncan 1972; Falk et al. 2023), and previous work has demonstrated that autistic and non‐autistic individuals differ in how often they display different types of socially‐relevant head movements, such as nods (García‐Pérez et al. 2007; Rifai et al. 2022). Additionally, head motion synchrony with a social partner distinguished autistic from non‐autistic individuals at above‐chance levels (Koehler et al. 2024). These differences in head motion synchrony in autistic and non‐autistic individuals may be linked to interaction outcomes, making it important to directly examine links between head motion synchrony and interaction outcomes like interaction enjoyment.

Synchrony in interactions involving autistic youth has been largely characterized in interactions between youth and parents, clinicians, or unfamiliar adults (Carnevali et al. 2024; Glass and Yuill 2023; McNaughton and Redcay 2020). However, peer interactions are characterized by different verbal and nonverbal features compared to conversations between youth and adults (Turkstra 2001). Furthermore, although autistic youth report particular challenges in peer interactions, such as perceptions of reduced peer interaction enjoyment and experiences of peer victimization (Cresswell et al. 2019; McNaughton et al. 2025), limited work has investigated synchrony in youth peer interactions (Glass and Yuill 2024; McNaughton et al. 2024; Stoit et al. 2011; Trevisan et al. 2021). These previous studies have focused on other aspects of synchrony like facial expressions (McNaughton et al. 2024) and structured cooperative motor tasks (Glass and Yuill 2024; Stoit et al. 2011; Trevisan et al. 2021). Therefore, characterizing synchrony in minimally structured peer interactions will provide important insight into the functioning of interpersonal synchrony in a context in which autistic youth report particular difficulty.

Middle childhood and adolescence has been proposed as a particularly notable period for investigating interaction dynamics between peers (Rauchbauer and Grosbras 2020). In this developmental period, peer relationships become an increasingly important source of social support for youth (Furman and Buhrmester 1992), as well as increasingly complex and intimate (Ladd 1999; Rubin et al. 2007). In turn, peer interactions also become increasingly difficult for autistic youth to navigate through adolescence as social demands and expectations increase with development (Cresswell et al. 2019). However, despite evidence that synchrony‐related processes may differ between youth and adults (Cook and Bird 2011), and between peer and non‐peer interactions (Ardizzi et al. 2014), there has been little research characterizing the presence of interpersonal motion synchrony in naturalistic contexts in youth peer relationships.

Computer vision approaches, such as automated approaches for tracking human body pose (Cao et al. 2021), provide rich opportunities for characterizing synchrony in developmental and neurodiverse populations. These computer vision approaches can identify and track human body positions in naturalistic settings without invasive markers or sensors, providing data on body position that is more objective and efficient than manual video coding methods. Additionally, these computer vision approaches may be especially beneficial for neurodiverse and developmental populations, as they avoid the use of wearable technology that may be impacted by participants’ sensory sensitivities or may influence participants’ motion. Computer vision approaches also provide opportunities to quantify head motion across a range of frequency bands, including high‐frequency, rapid nodding movements, and low‐frequency, slow head shifts. This may be particularly important for characterizing head motion synchrony, as the frequencies at which nodding head movements commonly occur do not necessarily display evidence of synchrony, while slower frequency head movements do display evidence of synchrony (Hale et al. 2020), and recent research has suggested that synchrony at different frequency bands may reflect different underlying mechanisms (Hamilton et al. 2025). In this way, computer vision approaches offer advantages to quantifying synchrony across frequency bands that are not captured by manual coding approaches.

Computer vision approaches have previously been used in research with autistic individuals (De Belen et al. 2020). These approaches have shed new insight into differences in head motion between autistic and neurotypical toddlers (Krishnappa Babu et al. 2023), children (Martin et al. 2018; Zhao et al. 2021), and adults (McDonald et al. 2023). Computer vision approaches have also been used to uncover interpersonal head motion dynamics in autistic children and familiar adult teachers (X. Chen et al. 2023). These previous findings demonstrate the feasibility of using computer vision to advance understanding of social dynamics in autism. Nonetheless, a gap remains in using these methods to quantify synchrony in peer interactions involving autistic and non‐autistic youth and relating synchrony to interaction outcomes.

Here we test the role of automatically quantified head motion synchrony in “getting‐to‐know‐you” conversations between peer dyads of autistic and non‐autistic youth. We test the hypothesis that automatically quantified head motion synchrony differs across the three dyad types: matched autistic, mismatched neurotype, and matched non‐autistic. We further test the hypothesis that synchrony predicts youth‐reported interaction enjoyment. We demonstrate significant evidence of automatically quantified head motion synchrony in naturalistic conversations between youth. Contrary to our hypothesis, we find that head motion synchrony does not differ across dyad types. Furthermore, we find that synchrony negatively predicts youth‐reported interaction enjoyment and positively predicts youth‐reported discomfort. These findings provide novel evidence on the presence and correlates of naturally occurring synchrony in neurodiverse youth peer interactions.

## 2 Methods

### 2.1 Participants

Youth were recruited from the Washington, D.C. metro area to participate in a lab‐based peer interaction as part of two separate studies. For the first study, autistic (*n* = 33) and neurotypical (*n* = 103) youth (aged 8–16 years) participated in a lab‐based peer interaction between Fall 2018 and Spring 2020. For the second study, autistic (*n* = 31) and non‐autistic (*n* = 69) youth (aged 11–15 years) participated in a lab‐based peer interaction between Summer 2022 and Summer 2025. Youth were recruited from previous participation in research, advertisements on Facebook, outreach at local events, or through the Simons Foundation Powering Autism Research (SPARK). We appreciate obtaining access to recruit participants through the SPARK research match on SFARI Base. All procedures were approved by the University of Maryland Institutional Review Board, and participants and their parents provided informed consent and assent.

All participants had a full‐scale IQ of 80 or higher based on administration of the Kaufman Brief Intelligence Test, 2^nd^ edition (KBIT‐2) (Kaufman and Kaufman 2004) and were native English speakers. For the first study, neurotypical participants were screened and excluded for any neurological or psychiatric disorders, or first‐degree relatives with schizophrenia or autism. For the second study, non‐autistic participants were screened and excluded for first‐degree relatives with schizophrenia or autism, but participants were not excluded for common co‐occurring conditions with autism (anxiety, depression, obsessive‐compulsive disorder, or ADHD). Because of the differences in exclusion procedures between the two studies, participants from the first study are referred to as “neurotypical”, while participants from the second study are referred to as “non‐autistic”. In the combined sample, participants are referred to as “non‐autistic” because both sets of exclusion criteria are represented.

For the first study, autistic participants had their autism diagnoses confirmed by administration of the Autism Diagnostic Observation Schedule, Second Edition (ADOS‐2; Lord et al. 2012) by a clinician who met research reliability criteria of administration and coding the ADOS‐2. For the second study, autistic participants had their autism diagnoses confirmed by the administration of the Autism Diagnostic Interview‐Revised (ADI‐R; Rutter et al., 2003) by a clinician who met research reliability criteria of administration and coding of the ADI‐R. Research reliability refers to meeting inter‐rater reliability of at least 80% on the ADOS‐2 and at least 90% on the ADI‐R with a trained clinician and coder.

The participants were paired into dyads corresponding to one of three dyad types: autistic participants paired with autistic participants (matched autistic dyads), autistic participants paired with non‐autistic participants (mismatched neurotype dyads), and non‐autistic participants paired with non‐autistic participants (matched non‐autistic dyads). All dyads consisted of participants of the same gender (parent‐reported for study 1, child‐reported for study 2) and similar chronological age (within 1 year or grade level). Dyads were excluded if they had previously met each other outside of the study context. One matched non‐autistic dyad was excluded for meeting this criterion, resulting in a sample of 117 dyads for analysis (66 matched non‐autistic dyads, 38 mismatched neurotype dyads, 13 matched autistic dyads). 224 participants were part of only one dyad. 10 participants were part of two dyads; these participants were part of a dyad in study 1 and then returned in study 2 and participated in a second dyad. Although dyad types were intended to be equal across dyad types, the COVID‐19 pandemic interrupted data collection. Therefore, comparisons with the smaller matched autistic dyad type should be considered preliminary due to the small sample size. Demographic information for the two study samples, including participants’ age, gender, race, ethnicity, and socioeconomic status, is provided in Table 1 (Study 1) and Table 2 (Study 2). The study 1 sample overlaps with a sample used in previously published analyses on smiling synchrony (*n* = 136) and theory of mind (*n* = 100) in peer interactions between autistic and neurotypical youth (McNaughton et al. 2024; Alkire et al. 2022). The study was not preregistered. No a priori power analysis was performed.

**TABLE 1:** Demographic information for study 1 sample (*n* = 136).

	Autistic (*n* = 33)	Neurotypical (*n* = 103)	Full sample (*n* = 136)
Mean age at session (range)+	13.3 (8.7–16.7)	12.6 (8.7–16.9)	12.8 (8.7–16.9)
Mean KBIT‐2 overall score (range)+	114 (80–141)	119 (87–146)	118 (80–146)
Mean ADOS‐2 module 3 overall total (Social affect + restricted and repetitive behaviors)	11 (7–16)	NA	NA
Parent‐reported gender^*^			
Female	6	52	58
Male	27	51	78
Race^*^			
Asian	1	1	2
Black or African American	0	20	20
Missing/Did not wish to report	3	10	13
More than one race	1	17	18
White	28	55	83
Ethnicity			
Hispanic/Latino	2	10	12
Not Hispanic/Latino	28	81	109
Missing/Did not wish to report	3	12	15
Highest Level of parental education			
Some College/AA degree	0	10	10
College degree	5	18	23
Some graduate school	4	13	17
Post‐graduate degree	21	53	74
Missing/Did not wish to report	3	9	12
Household income			
Less than US$35,000	0	4	4
US$35,000–$75,000	3	9	12
Over US$75,000	27	81	108
Missing/Did not wish to report	3	9	12

*Note*: The ADOS‐2 Module 4 was administered for one participant. That participant's overall total score is not included in the group average because the overall total algorithms between the two modules differ. Differences in distribution of demographic variables were tested between autistic and neurotypical groups using *t*‐tests or chi‐squared tests (+, *p*<0.10, ^*^ *p*<0.05).

**TABLE 2:** Demographic information for study 2 sample (*n* = 100).

	Autistic (*n* = 31)	Non‐autistic (*n* = 69)	Full sample (*n* = 100)
Mean age at session (range)+	13.8 (11.8–15.6)	13.4 (11.4–15.8)	13.5 (11.4–15.8)
Mean KBIT‐2 overall score (range)+	110 (81–135)	116 (90–142)	114 (81–142)
Participant‐reported gender^*^			
Female	3	27	30
Male	27	41	68
Genderfluid	1	1	2
Race			
Asian	1	1	2
Black or African American	1	9	10
Missing/Did not wish to report	1	2	3
More than one race	3	15	18
White	25	42	67
Ethnicity			
Hispanic/Latino	1	7	8
Not Hispanic/Latino	29	62	91
Missing/Did not wish to report	1	0	1
Highest level of parental education^*^			
Some College/AA degree	5	2	7
College degree	6	12	18
Some graduate school	4	4	8
Post‐graduate degree	15	51	66
Missing/Did not wish to report	1	0	1
Household income+			
Less than US$66,000	4	4	8
US$66,000–$100,000	6	3	9
US$100,000–$160,000	8	20	28
Over US$160,000	12	39	51
Missing/Did not wish to report	1	3	4

*Note*: Differences in distribution of demographic variables were tested between autistic and non‐autistic groups using *t*‐tests or chi‐squared tests (+, *p*<0.10, ^*^ *p*<0.05).

### 2.2 Peer Interaction Task and Post‐Interaction Questionnaires

Each dyad participated in a 5‐min getting‐to‐know‐you conversation as the first activity as part of a larger peer interaction (Study 1: 25‐min total interaction length, Study 2: 20‐min total interaction length). Data from the 5‐min getting‐to‐know‐you conversation was used for these analyses to focus on unstructured initial conversations and to harmonize data across the two studies.

For the 5‐min conversation, youth sat in chairs approximately 0.7 m across from each other in a room with a small, low table and three video cameras on one side. The experimenter told the youth that they would complete a few short tasks together, and that they should get to know each other before the experimenter came back in five minutes to explain the first task. The interactions were recorded using Canon Vixia HR F80 video cameras at a resolution of 1280 × 720 and 30 frames per second. For the first study, youth had no additional wearable technology. For the second study only, youth wore mobile eye‐tracking glasses attached to a phone that was stored in a fanny pack.

Following the face‐to‐face interaction, youth in both studies moved to separate rooms to complete activities independently, including a six‐item questionnaire describing their perceptions of the interaction (“Interaction Quality”; sum of six items with higher values indicating better quality) (Berry and Hansen 1996). Youth in study 1 also completed a sliding scale question measuring their desire to interact again (‘Desire to Interact Again’; −100 to 100 scale with very positive indicating strong desire to indicate with that partner again). Additional details on wording of the post‐interaction questionnaires is provided in Supplemental Information, and both measures have been previously used in studies of interaction quality in autistic and neurotypical youth (McNaughton et al. 2024). Participants in study 1 also completed additional questions on their discomfort following the interaction, which were summed into a four‐item composite with higher values indicating higher perceived discomfort during the interaction (see Supplemental Information for additional details on wording of items). Some participants did not complete one or more of the questionnaires, because the questions were added to the study protocol after the participants had completed their visit or because the participant chose to end their visit before completing the questions. Four participants in study 1 (*n* = 1 autistic, *n* = 3 non‐autistic) did not complete the interaction quality questionnaire (*n* = 4 because the questionnaire was added to the study protocol after participants completed their visit), 24 participants in study 1 (*n* = 11 autistic, *n* = 13 non‐autistic) did not complete the desire to interact again questionnaire (*n* = 20 because the questionnaire was added to the study protocol after participants completed their visit, *n* = 4 because participants chose to end their visit before completing the questionnaire), and four participants in study 1 (*n* = 1 autistic, *n* = 3 non‐autistic) did not complete the discomfort questions (*n* = 4 because the questionnaire was added to the study protocol after the participants completed their visit). Youth‐reported interaction quality and discomfort did not significantly differ by dyad type. Youth‐reported desire to interact again was significantly higher in matched non‐autistic dyads compared to mismatched neurotype dyads (*B* = 36.79, *t*(54.05) = 2.49, *p* = 0.02) and significantly higher in matched autistic dyads compared to mismatched neurotype dyads (*B* = 54.21, *t*(56.07) = 2.41, *p* = 0.02), while matched autistic and matched non‐autistic dyads did not significantly differ (*B* = 17.42, *t*(56.25) = 0.90, *p* = 0.37). These findings are consistent with previously reported analyses in an overlapping sample McNaughton et al. 2024).

Dyads were not informed by the research team about whether their peer interaction partner had an autism diagnosis or not. Some participants chose to self‐disclose their diagnosis during the interaction. A total of five autistic participants chose to self‐disclose: two autistic participants in mismatched neurotype dyads, and three autistic participants in matched autistic dyads, two of whom were in the same dyad.

### 2.3 OpenPose Analysis of Head Motion Coherence

**FIGURE 1:**
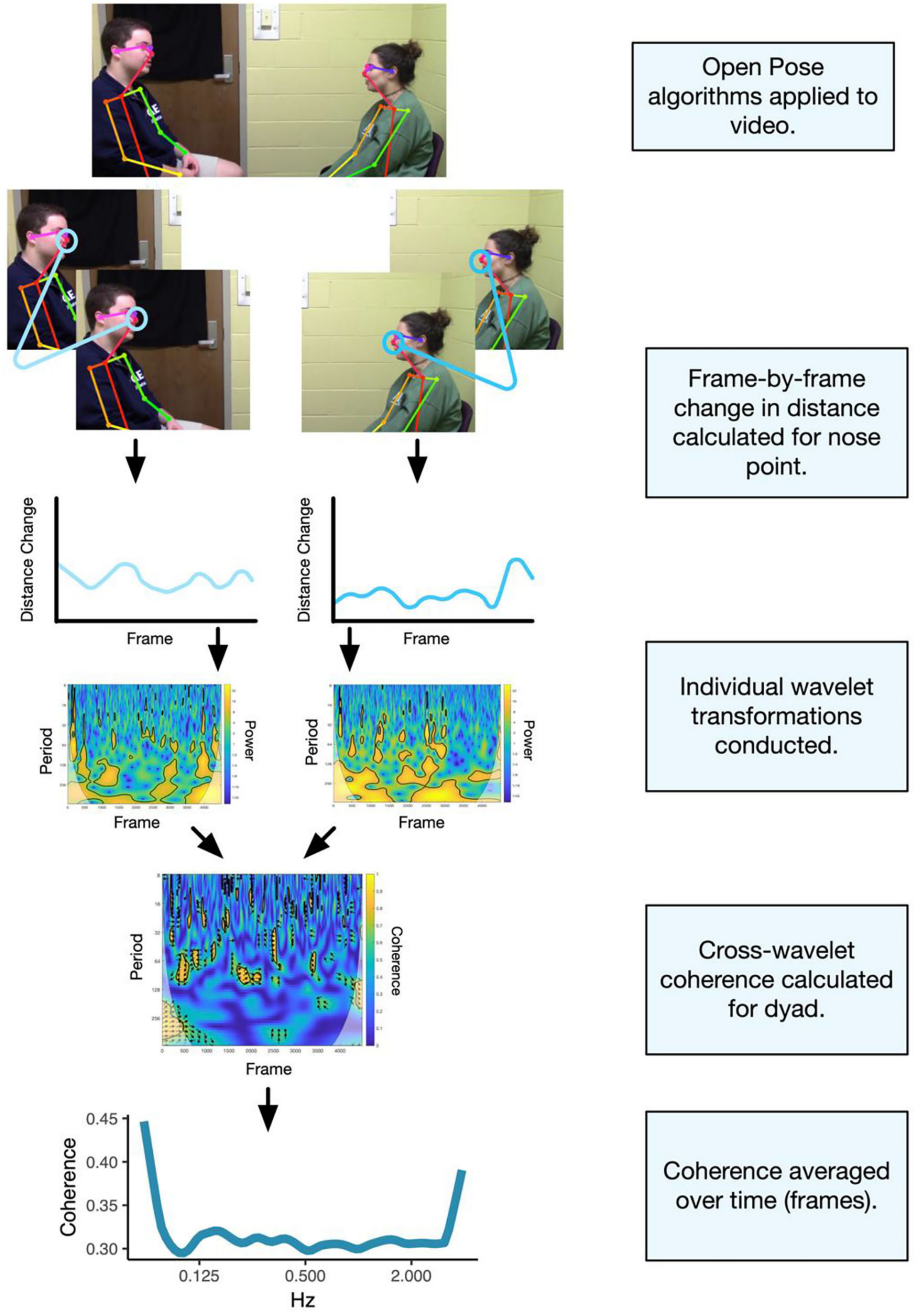
Analysis pathway for extraction of coherence values for example dyad. Nose point coordinates for each pair of participants were extracted using OpenPose algorithms (Cao et al. 2021). Separately for each participant, frame‐to‐frame change in nose coordinate position was transformed into a time series of head motion, which was then wavelet transformed for each participant. Cross‐wavelet coherence was calculated for each dyad. The cross‐wavelet coherence was averaged across the time series of the interaction, resulting in one coherence value per dyad per frequency. These coherence values were subsequently averaged into six frequency bands (0.0625–0.125 Hz, 0.125–0.25 Hz, 0.25–0.5 Hz, 0.5–1 Hz, 1–2 Hz, 2–4 Hz), to aid interpretation, resulting in one coherence value per frequency band per dyad. Individuals provided written informed consent for the use of their image in this article.

Video recordings of the five‐min getting‐to‐know‐you interaction were analyzed using OpenPose (Cao et al. 2021) software to extract x and y coordinates for 25 body parts, as well as the confidence for the algorithm's location estimate. This labeling process occurred for each member of the dyad for each frame of the video file. The first 9000 frames, corresponding to exactly five minutes at 30 frames per second, were analyzed for each interaction. Based on previous research identifying the importance of head motion synchrony in dyadic conversations (Hale et al. 2020), only the nose point for each participant was used for the present analysis to quantify head motion. Because video footage was obtained in a profile view, analyzing the nose point meant that nodding head motion in the pitch axis of motion was largely represented, consistent with previous work identifying significant evidence of pitch head motion synchrony (Hale et al. 2020). Figure 1 and Video S1 present the analysis workflow for a brief clip from an example dyad.

Data were processed if participants were within the video frame for at least 80% of the video and the interaction was at least five minutes/9000 frames. Data from 10 dyads (*n* = 1 matched autistic dyad, *n* = 5 mismatched dyads, *n* = 4 matched non‐autistic dyads) did not meet this threshold and were not processed further. Once processed, data were considered useable if at least 75% of frames were identified as having nose point confidence ratings for both participants above 0.80. The nose point confidence ratings were assigned on a 0 (lowest) to 1 (highest) scale by the computer algorithm, and the 0.80 cutoff was confirmed as representing a cutoff after which data quality decreased based on comparisons to a subset of files annotated by a human coder. Data from five dyads (*n* = 2 matched autistic dyads, *n* = 1 mismatched dyad, *n* = 2 matched non‐autistic dyads) did not meet this threshold. Data were subject to further manual inspection, and two matched non‐autistic dyads were excluded based on discovery of artifacts. Following these exclusions, 100 dyads were included in analyses across the two studies (study 1: *n* = 5 matched autistic dyads, *n* = 16 mismatched dyads, *n* = 38 matched non‐autistic dyads, study 2: *n* = 5 matched autistic dyads, *n* = 16 mismatched dyads, *n* = 20 matched non‐autistic dyads).

Frame‐by‐frame data were concatenated to form time series of nose position for each participant using in‐house scripts written in R (available on OSF: https://osf.io/tq7su/). Missing data, which occurred due to occlusion of the nose or the nose being outside of the video frame, were imputed using stine imputation to reflect the non‐linear nature of the position data. An average of 40 frames were imputed for each participant (min = 0, max = 1198), with an average maximum consecutive frames imputed per participant of 13 (min = 0, max = 436) across the 9000 frame time series. The time series representing the x and y nose coordinate positional data were then transformed into a frame‐by‐frame change in distance metric using Euclidean distance, yielding one time series of head motion for each participant. The absolute difference between each sequential point on this time series was summed to create a metric of overall motion that was included in analyses as a covariate.

Synchrony was quantified using cross‐wavelet coherence, which allows the time‐frequency nature of head motion to be respected, such that synchrony between slower low frequency head movements (e.g. gradual shifts) is disentangled from synchrony between faster high frequency head movements (e.g. fast nods (Hale et al. 2020)). The head motion time series for each participant was subjected to a wavelet transformation with parameters previously used for analyzing social coordination data (Morlet wavelet, w = 6; (Hale et al. 2020)). Within the dyads, the cross‐wavelet coherence was calculated at 73 frequencies in the 0.0625–4 Hz frequency range. This 0.0625 Hz‐4 Hz frequency range is in line with the frequency (30 Hz) and length (150 seconds per interaction half) of data collection in the present study and consistent with previously identified frequencies of interest for head motion synchrony (Hale et al. 2020). This output was then averaged into six frequency bands to aid interpretation and direct comparison with previous work (0.0625–0.125 Hz, 0.125–0.25 Hz, 0.25–0.5 Hz, 0.5–1 Hz, 1–2 Hz, 2–4 Hz (Fujiwara and Yokomitsu 2021)), resulting in one coherence value per frequency band per dyad.

### 2.4 Statistical Analysis

To establish evidence of group‐level coherence, data from true pairs were statistically compared to time‐scrambled data from those same pairs. That is, wavelet transformations from the first half of individual A's true time series and the first half of individual B's true time series, as well as wavelet transformations from the second half of individual A's true time series and the second half of individual B's true time series were each subjected to a cross‐wavelet transformation and averaged to extract a single “true” coherence value. Wavelet transformations from the first half of individual A's time series and the second half of individual B's time series, as well as from the second half of individual A's time series and the first half of individual B's time series were subjected to a cross‐wavelet transformation and then averaged to extract a single “pseudo” coherence value. Six paired *t*‐tests were performed to compare data from these true pairs and pseudo pairs, one test for each of the frequency bands, with false discovery rate correction for multiple comparisons across 6 frequency bands (0.0625–0.125 Hz, 0.125–0.25 Hz, 0.25–0.5 Hz, 0.5–1 Hz, 1–2 Hz, 2–4 Hz). Analytic results for the comparison between true and pseudo coherence in the original 73 frequency values obtained from the wavelet analysis before averaging into the six frequency bands are presented in Supplemental Information (Figure S2).

For the remaining analyses on dyad type differences in synchrony and relations between synchrony and interaction enjoyment, we report coherence in the frequency band(s) that showed significant differences between true and pseudo pairs. These analyses are confirmatory analyses testing our primary hypotheses. We provide details on frequency bands that did not display evidence of significant synchrony in the Supplemental Information for completeness of reporting.

To assess dyad type differences in synchrony, linear models were performed with dyad type (matched autistic, mismatched neurotype, matched non‐autistic) as the predictor of interest and coherence in a given frequency band as the outcome of interest (model details in ‘Dyad Type Differences in Head Motion Synchrony Supplemental Information **’**). Age, gender, data quality (proportion of frames missing from analysis/below a confidence level of 0.8), overall motion, and study (study 1 or 2) were included as covariates based on potential for confounds. Results were robust to inclusion/exclusion of covariates. Follow‐up Bayesian ANOVAs were performed in JASP v0.95.1 (JASP Team 2025) to quantify the evidence for and against dyad type differences (Van Den Bergh et al. 2020; Van Doorn et al. 2021).

To assess relations between synchrony and interaction outcomes (interaction quality, desire to interact again, discomfort), multilevel models were constructed treating youth‐reported interaction enjoyment as nested within dyads, and intercepts were modeled as randomly varying across dyads. The discomfort outcome was square‐root transformed to better meet normality assumptions. Coherence in a given frequency band was the predictor of interest, and time‐scrambled pseudocoherence, age, gender, dyad type (matched autistic/mismatched neurotype/matched non‐autistic), data quality (proportion of frames missing from analysis/below a confidence level of 0.8), overall motion, and study (study 1 or 2, for the interaction quality outcome that was collected across both studies) were included as covariates of no interest. Results were robust to inclusion/exclusion of covariates. Each outcome (interaction quality, desire to interact again, discomfort) was treated as its own family of tests as prior work has shown these outcomes may reflect separable constructs (Berridge et al. 2009). Analyses were performed in R (R Core Team 2020) using the lmerTest package (Kuznetsova et al. 2017).

## 3 Results

### 3.1 Evidence of Significant Head Motion Synchrony in Naturalistic Peer Interactions

**FIGURE 2:**
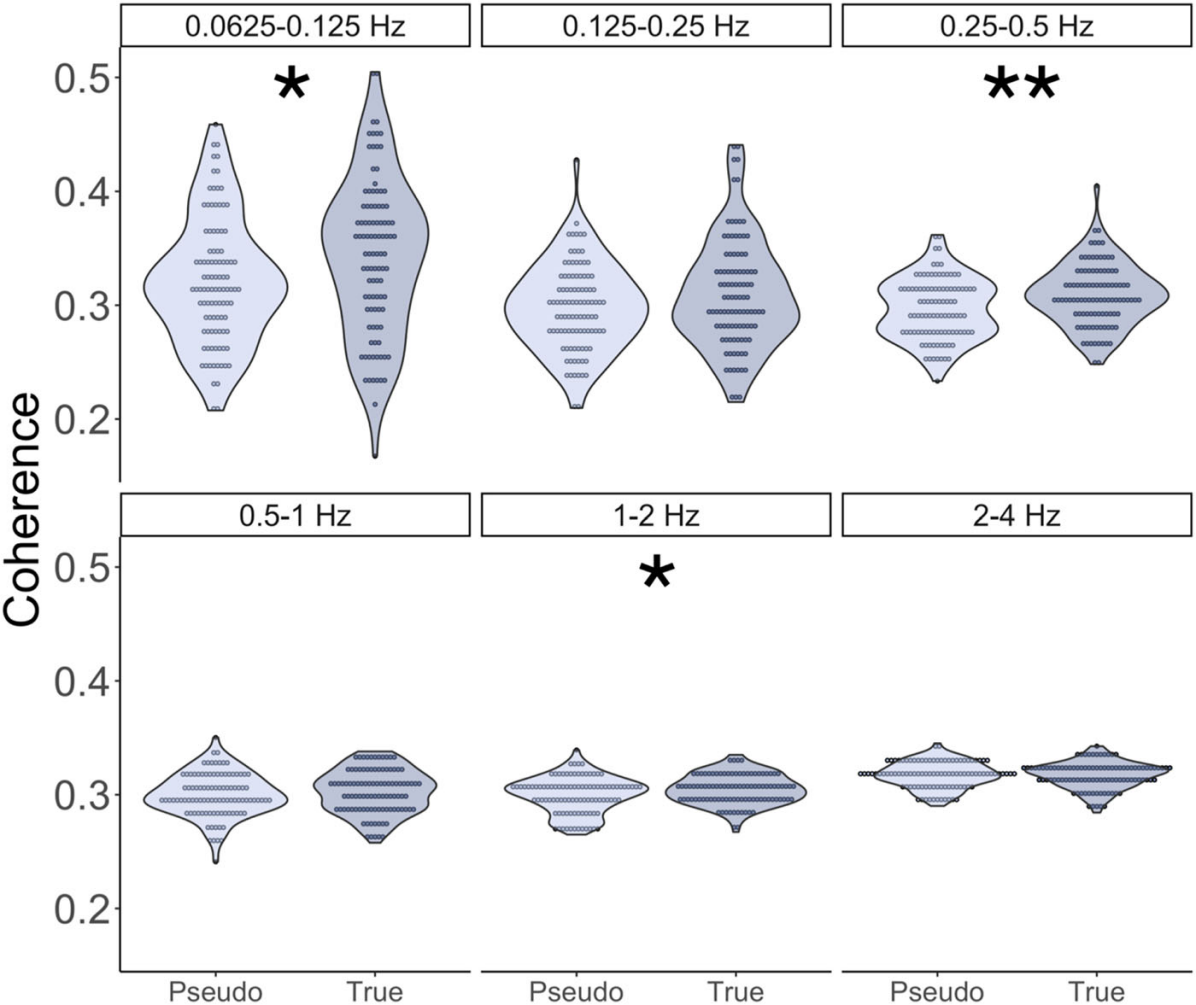
Evidence of significant head motion synchrony in naturalistic conversations (*n* = 100 dyads). True dyads had significantly greater coherence (**, *p*_corrected_<0.01, *, *p*_corrected_<0.05) in the 0.0625–0.125, 0.25–0.5 Hz, and 1–2 Hz frequency bands compared to time‐scrambled pseudo dyads.

To test for evidence of significant head motion synchrony in naturalistic peer interactions, we compared coherence for true dyads and time‐swapped pseudo dyads using paired *t*‐tests for each of the six coherence bands across all 100 dyads, with false discovery rate correction applied for six coherence bands. There was significant evidence of coherence in the 0.0625–0.125 Hz (*t*(99) = 2.77, *p*_corrected_ = 0.02), 0.25 Hz–0.5 Hz (*t*(99) = 3.66, *p*_corrected_ = 0.002), and 1–2 Hz (*t*(99) = 2.51, *p*_corrected_ = 0.03) frequency bands (Figure 2). There was no significant evidence of coherence in the 0.125–0.25 Hz frequency band (*t*(99) = 2.02, *p*_corrected_ = 0.07), 0.5–1 Hz (*t*(99) = 1.14, *p*_corrected_ = 0.31), or 2–4 Hz (*t*(99) = 0.18, *p*_corrected_ = 0.86) frequency bands.

### 3.2 Dyad Type Differences in Head Motion Synchrony

**FIGURE 3:**
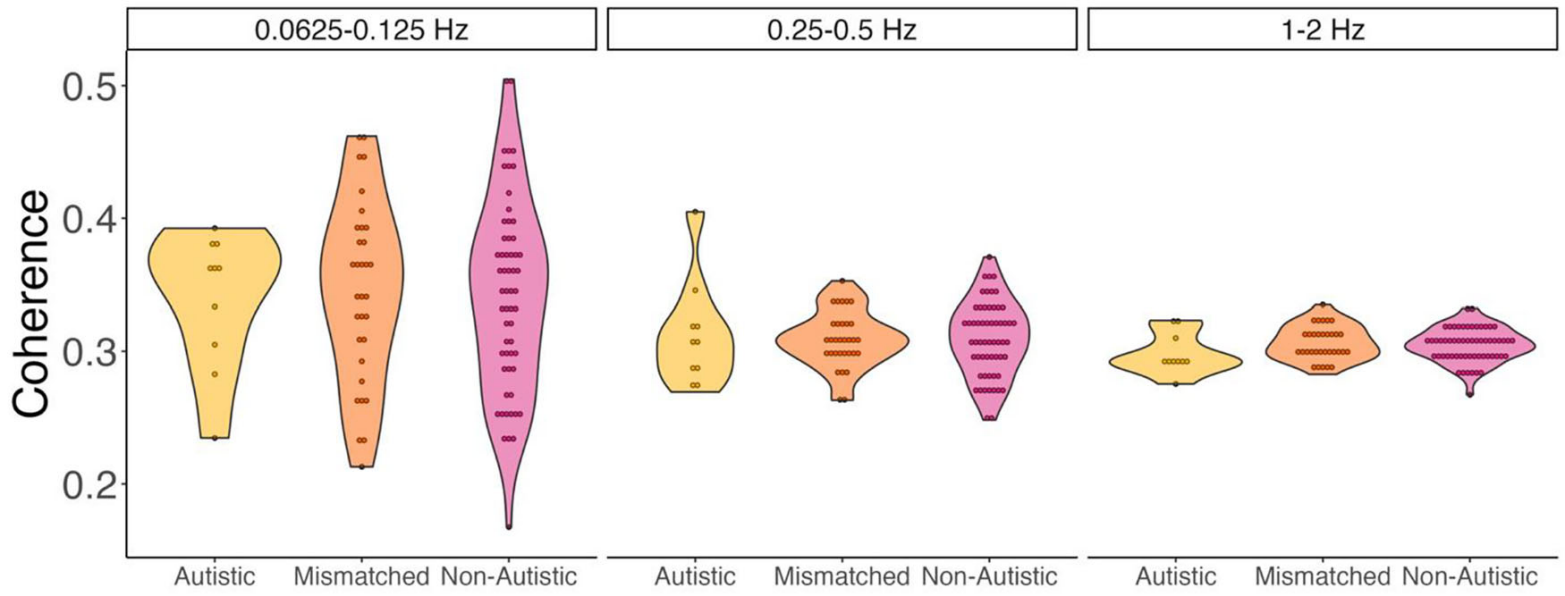
Lack of significant dyad type differences in head motion synchrony in the frequency bands that demonstrated above‐chance coherence.

To test for dyad type differences in head motion synchrony, we conducted three linear models for each of the frequency bands with significant evidence of coherence as the outcome and dyad type (matched autistic, mismatched neurotype, matched non‐autistic) as a predictor of interest. There were no significant differences in head motion synchrony between dyad types in the three frequency bands that demonstrated significant evidence of above‐chance coherence: 0.0625–0.125 Hz, 0.25–0.5 Hz, or 1–2 Hz (Figure 3; Tables S1–3). Follow‐up Bayesian analyses to quantify support for and against an effect of dyad type revealed moderate evidence against an effect of dyad type in the frequency bands that demonstrated evidence of above‐chance coherence (Table S7).

### 3.3 Relations Between Head Motion Synchrony and Interaction Enjoyment

To test for relations between head motion synchrony and interaction enjoyment, we conducted multilevel models with head motion synchrony in a given frequency band as the predictor of interest. For the frequency bands with above‐chance evidence of coherence, separate models were constructed for each of the three outcomes of interest, which were administered at different studies: interaction quality (including participants from study 1 and 2, study included as covariate), desire to interact again (participants from study 1), and discomfort (participants from study 1). For the six‐item interaction quality outcome, head motion synchrony in the 0.0625–0.125, 0.25–0.5 Hz, and 1–2 Hz bands did not significantly predict interaction quality (Table S8,S11).

**FIGURE 4:**
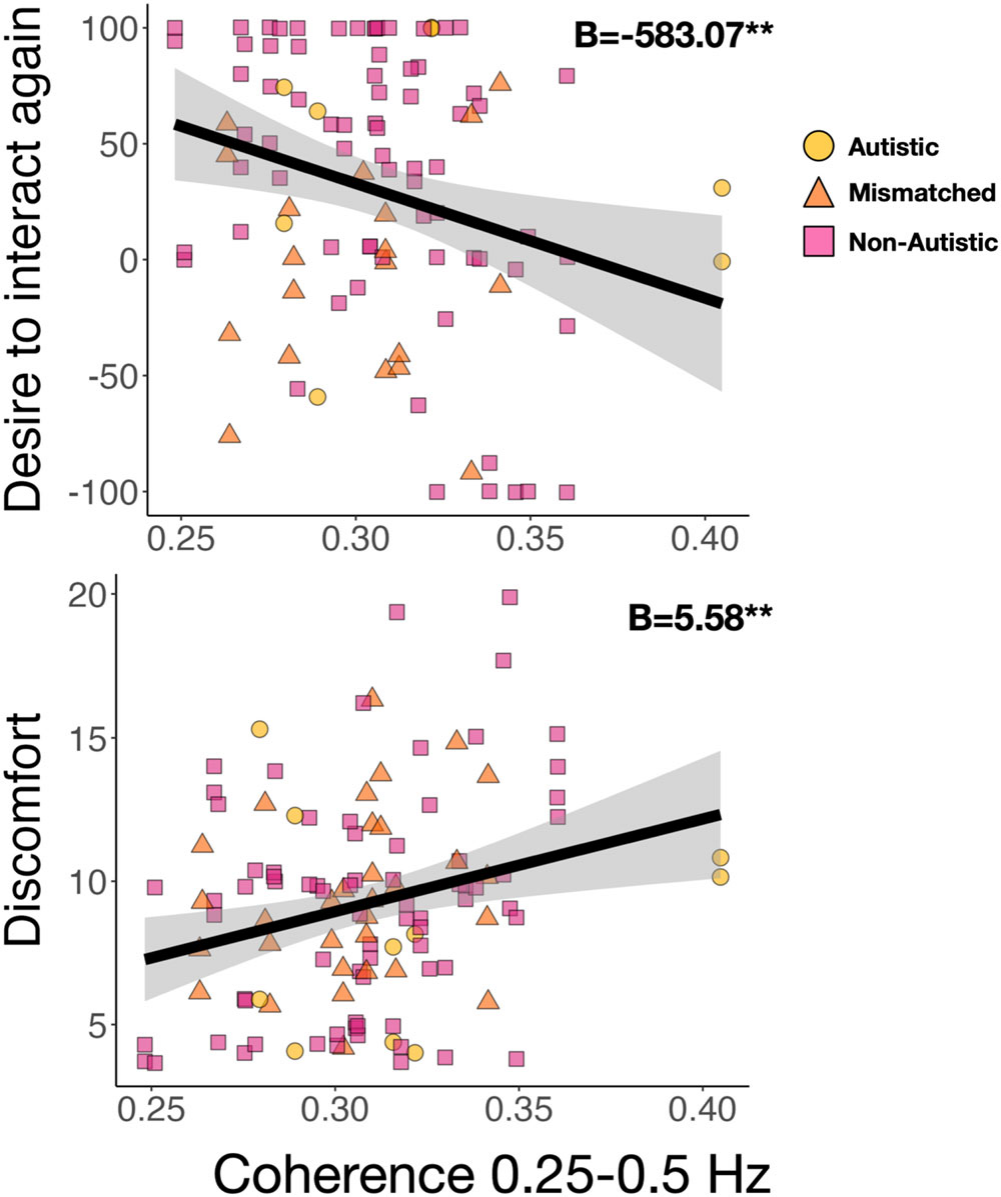
Relations between head motion synchrony in the 0.25–0.5 Hz coherence band and interaction outcomes in matched autistic, mismatched, and matched non‐autistic dyads. (A) Head motion synchrony significantly negatively predicted desire to interact again. (B) Head motion synchrony significantly positively predicted discomfort in the interaction. Circle points represent a participant in a matched autistic dyad, triangle points represent a participant in a mismatched dyad, and square points represent a participant in a matched non‐autistic dyad. Estimates are unstandardized betas (**, *p*<0.01). The shaded area around the regression lines represents the 95% confidence interval.

For the desire to interact again sliding scale, head motion synchrony in the 0.25–0.5 Hz frequency band significantly negatively predicted desire to interact again (*B* = −583.07, *t*(40.57) = −2.91, *p* = 0.01; Table S9), such that youth reported less desire to interact again with their peer partner after interactions with greater head motion synchrony (Figure 4A). Head motion synchrony in the 0.0625–0.125 and 1–2 Hz bands did not significantly predict desire to interact again (Table S12). Findings remained significant with false discovery rate correction for the three above‐chance coherence bands.

For the discomfort measure, head motion synchrony in the 0.25–0.5 Hz (*B* = 5.58, *t*(49.07) = 2.72, *p* = 0.01; Table S10) frequency band significantly positively predicted discomfort, such that youth reported experiencing greater discomfort with their peer partner during interactions with greater head motion synchrony (Figure 4B). Head motion synchrony in the 0.0625–0.125 and 1–2 Hz bands did not significantly predict discomfort (Table S13). Findings remained significant with false discovery rate correction for the three above‐chance coherence bands.

Exploratory models were also constructed including statistical interaction terms between dyad type and head motion synchrony to predict interaction outcomes. There were no significant statistical interactions between dyad type and head motion in predicting interaction quality, desire to interact again, or discomfort that remained after correction for multiple comparisons across frequency bands (*p*s>0.05). Models were also constructed for relations between synchrony and interaction outcomes in each of the frequency bands that did not display significant above‐chance evidence of synchrony (0.125–0.25 Hz, 0.5–1 Hz and 2–4 Hz) for completeness of reporting (Tables S11–S13).

## 4 Discussion

Using an automated computer vision approach, we investigated the presence of head motion synchrony in naturalistic conversations between youth, as well as relations between synchrony and interaction outcomes such as interaction enjoyment and discomfort. We found evidence for significant head motion synchrony in getting‐to‐know‐you conversations involving autistic and non‐autistic youth. We found that synchrony did not differ across dyad types (matched autistic/matched non‐autistic/mismatched neurotype), but that dyadic differences in synchrony did predict youth's perceptions of interaction enjoyment. Specifically, youth reported decreased desire to interact again and increased discomfort in interactions with greater head motion synchrony. Together, these findings refine understanding of the role of naturally occurring synchrony in interactions between youth, underscoring that synchrony may not always benefit youth's perceptions of a social interaction in contrast with previous work with experimentally induced synchrony.

### 4.1 Head Motion Synchrony Naturally Occurs at Above‐Chance Levels in Youth Conversations

We found evidence of above‐chance synchrony in frequency bands ranging from 0.0625–2 Hz. This finding of significant synchrony in more gradual, lower frequency head movements (in contrast to high frequency movements) is consistent with previous findings from adults engaged in conversational tasks (Falk et al. 2023; Hale et al. 2020), supporting the existence of spontaneous social coordination across development (Rauchbauer and Grosbras 2020).

Because of the camera location and selection of the nose point for analysis, the analyzed head motion time series largely represent nodding and activity in the “pitch” axis of head motion. Previous work has suggested that these low frequency nods may be indicative of gaze following behaviors (Falk et al. 2023), such that low frequency synchrony could indicate gaze synchrony between partners. Overall, our results reinforce the idea that synchrony occurs in specific types of head movements, not across all head movements.

The use of computer vision methods to capture significant evidence of synchrony in similar frequency bands to previous work with wearable devices (Falk et al. 2023; Hale et al. 2020) supports the use of these methods for quantifying naturally occurring synchrony. Non‐invasive computer vision methods may be particularly important for populations with sensory sensitivities, such as autistic individuals, and research questions that would be affected by participants being aware of the wearable devices or feeling them impact their motion patterns. Furthermore, because this analytic pipeline uses video recordings that are commonly obtained in research and clinical settings, our approach is economical and practical for use on pre‐existing datasets, presenting practical advantages for many developmental science questions of interest.

### 4.2 Dyad Types Do Not Differ in Head Motion Synchrony

We found no evidence for differences in head motion synchrony across dyad types (matched autistic, matched non‐autistic, mismatched neurotype). Previous findings have pointed to motion synchrony differences between mismatched neurotype dyads and matched non‐autistic dyads (Carnevali et al. 2024; Glass and Yuill 2023). Specifically, previous work investigating conversations between adults has demonstrated reduced head and whole‐body motion synchrony in mismatched neurotype dyads compared to matched non‐autistic dyads (Georgescu et al. 2020; Koehler et al. 2022), and previous work investigating social interactions between children and adult experimenters has found reduced head and whole‐body synchrony for autistic children interacting with experimenters compared to non‐autistic children interacting with experimenters during structured tasks (Noel et al. 2018; Zadok et al. 2022).

One important difference between our work and prior work is that our study is the first to investigate head motion synchrony in youth peer interactions. Youth display different speaker and listener behaviors when interacting with peers compared to adults (Turkstra 2001), highlighting the importance of assessing social interactions in the social context of interest. Future research could more directly test differences between adult‐child and peer interactions by having children engage in separate interactions with both a peer and an adult partner, enabling within‐subjects comparisons of synchrony in these two distinct social environments.

Another difference is that, in the present study, youth engaged in an unstructured conversation, while other research has used more structured tasks (Noel et al. 2018; Zadok et al. 2022). Task context may impact synchrony. One recent study examined unstructured play interactions between children and an adult experimenter and found that dyad type differences in head motion synchrony varied based on whether there was joint engagement in the interaction (X. Chen et al. 2023), highlighting that within‐interaction dynamics play an important role in synchrony differences. Previous research in adults has also demonstrated that head motion synchrony differs across tasks (Falk et al. 2023). Interestingly, previous research using an unstructured conversation similar to our approach also did not find dyad type differences in synchrony (Efthimiou et al. 2025), while research using directed discussion prompts or semi‐structured conversations did find dyad type differences in synchrony (Georgescu et al. 2020).

Finally, previous work on synchrony has utilized windowed cross correlations to quantify the presence and degree of synchrony. Windowed cross‐correlations focus on the time‐domain, such that they identify synchrony across the time series as a whole rather than within different frequency bands (Fujiwara and Daibo 2016), and have previously provided valuable insight into the presence of dyad type differences in synchrony (Georgescu et al. 2020). Furthermore, parameter choices in the windowed cross correlation, such as the amount of lag in the time domain at which changes can be quantified as “synchronous” (e.g. 5 seconds (Georgescu et al. 2020; Koehler et al. 2022)), influence how synchrony is identified within a time series. The absence of differences using cross‐wavelet methods of synchrony quantification could reveal that underlying synchrony differences between autistic and neurotypical dyads are not strong in the frequency dimension, highlighting how different approaches to quantifying synchrony provide a more complete picture of the nature of synchrony differences between autistic and neurotypical individuals.

### 4.3 Head Motion Synchrony Predicts Decreased Interaction Enjoyment and Increased Discomfort

We identified negative relations between head motion synchrony and interaction enjoyment, which contrasts with previous findings of positive social outcomes (increased rapport, closeness, etc.) in response to experimentally‐induced synchrony, for a range of body motions (Hove and Risen 2009; Mogan et al. 2017; Rennung and Göritz 2016; Vicaria and Dickens 2016). Our findings further contrast with a previous study identifying positive relations between rapport and spontaneous motion synchrony in a face‐to‐face conversation, although this study investigated whole‐body synchrony, not head synchrony specifically (Fujiwara et al. 2020). An advantage of our approach using cross‐wavelet coherence is that it points to specific synchrony patterns that link to reduced enjoyment, as we find these relations specifically in low frequency head movements, not in high frequency rapid nods, and future work could supplement these findings with additional manual coding of specific movements.

One interpretation of these findings could be that our metric of synchrony captures markers of synchronized discomfort, which then link to reduced enjoyment. We found relations between a composite of youth‐reported discomfort in the interaction and increased synchrony in the interaction. In the getting‐to‐know‐you context with an unfamiliar peer, higher synchrony in low frequency bands could capture aspects of synchronized head motion like mutual “cringes” of discomfort, shared awkward laughing, or synchronized gazing at other parts of the room, which then relate to increased youth‐reported discomfort. Future work that measures moment‐to‐moment youth‐reported discomfort in the interaction could better identify these markers of discomfort and link them to synchrony over the time series of the interaction.

Our findings also fit in with a growing body of work emphasizing that more synchrony is not always better (Mayo and Gordon 2020). Instead, rapport may be influenced by other factors like divergence, novelty, and exploration, rather than exclusively synchrony (Ravreby et al. 2022; Speer et al. 2024). Furthermore, the role of synchrony may depend on context, such that reduced synchrony may help with problem solving or indicate conversation ease. In a problem solving task, weaker full‐body synchrony in interacting adult dyads predicted better task performance (Abney et al. 2015). Increased lower‐frequency head motion synchrony was associated with conversations with greater background noise, which could be interpreted as a marker of conversation difficulty (Hadley and Ward 2021). The importance of synchrony in context is also seen in the developmental origins of synchrony; that is, moderate, rather than high or low, synchrony in mother‐child interactions at 4 months predicts secure attachment at 12 months (Beebe et al. 2010). Together, these studies emphasize roles for reduced synchrony in relationship development, conversational ease, problem solving, and novelty, all of which may support links between reduced synchrony and increased interaction enjoyment.

From this variety of perspectives, synchrony emerges not as a universal advantage, but as a marker with differential impacts across contexts. In this context of a brief getting‐to‐know‐you interaction between previously unacquainted youth, we found that head motion synchrony is associated with less interaction enjoyment and more discomfort. Our study highlights the importance of investigating social phenomena in naturalistic contexts and directly linking features of interactions to youth reports, as findings from experimental inductions of synchrony may not generalize to the phenomenon of naturally occurring synchrony. Because automated approaches are increasingly deployed to identify differences between interactions involving autistic and non‐autistic individuals, it important to consider the meaning of the features and how they relate back to participants’ experiences of the interaction. This validation of these conversational features will lead to the best understanding of youth peer interactions for autistic and non‐autistic youth.

### 4.4 Limitations and Future Directions

Given the small sample of matched autistic dyads, findings including this dyad type should be viewed as preliminary. Previous work has identified reduced motion synchrony between matched autistic and matched non‐autistic dyads (Georgescu et al. 2020; Stoit et al. 2011), therefore our null findings of differences between matched autistic and matched non‐autistic dyads may reflect the fact that the study was underpowered to identify differences. Including larger samples of matched autistic dyads in future work is crucial to understand autistic youth sociality across partner types, as there are differences in interaction enjoyment and interaction features between matched autistic and mismatched neurotype youth peer interactions (Y.‐L. Chen et al. 2021; McNaughton et al. 2024).

Future work could also use further approaches to quantify social interaction dynamics. For example, in line with recent theoretical calls to consider the dynamics of increased and reduced synchrony across the time course of the interaction, measures of metastability could be used to better calculate flexibility or entrance in and out of synchrony (Mayo and Gordon 2020; Tognoli et al. 2018). Additionally, future work could also take into consideration the multimodal nature of social interactions by integrating head motion synchrony with other constructs (e.g. turn taking, gaze etc.) to more fully characterize conversation dynamics (Fauviaux et al. 2024). These approaches will provide a richer characterization of social dynamics in peer interactions between autistic and non‐autistic youth, with further implications for understanding characteristics of interactions related to enjoyment and discomfort.

## 5 Conclusion

We demonstrate the application of a computer vision approach to non‐invasively measure head motion synchrony in youth peer interactions. We do not find evidence of differences in synchrony across the dyad types, and we further present novel evidence linking increased synchrony to reduced interaction enjoyment and increased discomfort. Our findings add additional nuance to understanding the correlates of naturally occurring synchrony in peer interactions, providing evidence that increased synchrony is not always associated with interaction enjoyment. These findings highlight the importance of directly linking interaction features to youth report of interaction enjoyment and underscore opportunities to further understand how synchrony impacts social interactions across development in context‐dependent ways.

## Author Contributions

**Kathryn A. McNaughton**: conceptualization, data curation, formal analysis, funding acquisition, methodology, software, project administration, visualization, investigation, writing – original draft, writing – review and editing. **Elizabeth Redcay**: conceptualization, funding acquisition, methodology, project administration, resources, supervision, writing – review and editing. **Heather A. Yarger**: conceptualization, investigation, project administration, resources, writing – review and editing.

## Funding

Research reported in this publication was supported by the National Institute of Mental Health of the National Institutes of Health under Award Nos. R01MH107441, R01MH125370, and F31MH127781. The content is solely the responsibility of the authors and does not necessarily represent the official views of the NIH.

## Ethics Statement

All procedures were approved by the University of Maryland Institutional Review Board and were conducted in accordance with the Declaration of Helsinki and US Federal Policy for the Protection of Human Subjects. Participants and their parents provided informed consent and assent.

## Conflicts of Interest

The authors declare no conflicts of interest.

## Supporting information




**Supporting Information**: desc70285‐sup‐0001‐SuppMat.docx

**Video**: analytic_demo_video.mov

## Data Availability

The data that support the findings described in this manuscript are available on the Open Science Framework: https://osf.io/tq7su.

## References

[desc70285-bib-0001] Abney, D. H. , A. Paxton , R. Dale , and C. T. Kello . 2015. “Movement Dynamics Reflect a Functional Role for Weak Coupling and Role Structure in Dyadic Problem Solving.” Cognitive Processing 16, no. 4: 325–332. 10.1007/s10339-015-0648-2. 25757891

[desc70285-bib-0073] Alkire, D. , K. A. McNaughton , H. A. Yarger , D. Shariq , and E. Redcay . 2022. “Theory of mind in naturalistic conversations between autistic and typically developing children and adolescents.” Autism 27, no. 2: 472–488. 10.1177/13623613221103699. 35722978 PMC9763550

[desc70285-bib-0002] Alsarrani, A. , R. F. Hunter , L. Dunne , and L. Garcia . 2022. “Association Between Friendship Quality and Subjective Wellbeing Among Adolescents: A Systematic Review.” BMC Public Health [Electronic Resource] 22, no. 1: 2420. 10.1186/s12889-022-14776-4. 36564745 PMC9784006

[desc70285-bib-0003] Ardizzi, M. , M. Sestito , F. Martini , M. A. Umiltà , R. Ravera , and V. Gallese . 2014. “When age Matters: Differences in Facial Mimicry and Autonomic Responses to Peers' Emotions in Teenagers and Adults.” PLoS ONE 9, no. 10: e110763. 10.1371/journal.pone.0110763. 25337916 PMC4206508

[desc70285-bib-0004] Ayache, J. , A. Connor , S. Marks , et al. 2021. “Exploring the “Dark Matter” of Social Interaction: Systematic Review of a Decade of Research in Spontaneous Interpersonal Coordination.” Frontiers in Psychology 12: 718237. 10.3389/fpsyg.2021.718237. 34707533 PMC8542929

[desc70285-bib-0005] Beebe, B. , J. Jaffe , S. Markese , et al. 2010. “The Origins of 12‐Month Attachment: A Microanalysis of 4‐Month Mother–Infant Interaction.” Attachment & Human Development 12, no. 1–2: 3–141. 10.1080/14616730903338985. 20390524 PMC3763737

[desc70285-bib-0006] Berridge, K. C. , T. E. Robinson , and J. W. Aldridge . 2009. “Dissecting Components of Reward: ‘Liking’, ‘Wanting’, and Learning.” Current Opinion in Pharmacology 9, no. 1: 65–73. 10.1016/j.coph.2008.12.014. 19162544 PMC2756052

[desc70285-bib-0007] Berry, D. S. , and J. S. Hansen . 1996. “Positive Affect, Negative Affect, and Social Interaction.” Journal of Personality and Social Psychology 71, no. 4: 796–809. 10.1037/0022-3514.71.4.796.

[desc70285-bib-0008] Cao, Z. , G. Hidalgo , T. Simon , S.‐E. Wei , and Y. Sheikh . 2021. “OpenPose: Realtime Multi‐Person 2D Pose Estimation Using Part Affinity Fields.” IEEE Transactions on Pattern Analysis and Machine Intelligence 43, no. 1: 172–186. 10.1109/TPAMI.2019.2929257. 31331883

[desc70285-bib-0009] Carnevali, L. , I. Valori , G. Mason , G. Altoè , and T. Farroni . 2024. “Interpersonal Motor Synchrony in Autism: A Systematic Review and Meta‐Analysis.” Frontiers in Psychiatry 15: 1355068. 10.3389/fpsyt.2024.1355068. 38439792 PMC10909819

[desc70285-bib-0010] Chen, X. , J. Chen , M. Liao , and G. Wang . 2023. “Early Onset of Impairments of Interpersonal Motor Synchrony in Preschool‐Aged Children With Autism Spectrum Disorder.” Journal of Autism and Developmental Disorders 53, no. 6: 2314–2327. 10.1007/s10803-022-05472-8. 35303748

[desc70285-bib-0011] Chen, Y.‐L. , L. L. Senande , M. Thorsen , and K. Patten . 2021. “Peer Preferences and Characteristics of Same‐Group and Cross‐Group Social Interactions Among Autistic and Non‐Autistic Adolescents.” Autism 25, no. 7: 1885–1900. 10.1177/13623613211005918. 34169757 PMC8419288

[desc70285-bib-0012] Cirelli, L. K. 2018. “How Interpersonal Synchrony Facilitates Early Prosocial Behavior.” Current Opinion in Psychology 20: 35–39. 10.1016/j.copsyc.2017.08.009. 28830004

[desc70285-bib-0013] Cook, J. , and G. Bird . 2011. “Social Attitudes Differentially Modulate Imitation in Adolescents and Adults.” Experimental Brain Research 211, no. 3–4: 601–612. 10.1007/s00221-011-2584-4. 21336831 PMC3102210

[desc70285-bib-0014] Cresswell, L. , R. Hinch , and E. Cage . 2019. “The Experiences of Peer Relationships Amongst Autistic Adolescents: A Systematic Review of the Qualitative Evidence.” Research in Autism Spectrum Disorders 61: 45–60. 10.1016/j.rasd.2019.01.003.

[desc70285-bib-0015] De Belen, R. A. J. , T. Bednarz , A. Sowmya , and D. Del Favero . 2020. “Computer Vision in Autism Spectrum Disorder Research: A Systematic Review of Published Studies From 2009 to 2019.” Translational Psychiatry 10, no. 1: 333. 10.1038/s41398-020-01015-w. 32999273 PMC7528087

[desc70285-bib-0016] Duncan, S. 1972. “Some Signals and Rules for Taking Speaking Turns in Conversations.” Journal of Personality and Social Psychology 23, no. 2: 283–292. 10.1037/h0033031.

[desc70285-bib-0017] Efthimiou, T. N. , C. E. Wilks , S. Foster , et al. 2025. “Social Motor Synchrony and Interactive Rapport in Autistic, Non‐Autistic, and Mixed‐Neurotype Dyads.” Autism 13623613251319585. 10.1177/13623613251319585. 39994178

[desc70285-bib-0018] Falk, P. , R. Cañigueral , J. A. Ward , and A. F. D. C. Hamilton . 2023. Head Nodding and Hand Coordination Across Dyads in Different Conversational Contexts. 10.21203/rs.3.rs-3526068/v1.

[desc70285-bib-0019] Fauviaux, T. , L. Marin , M. Parisi , R. Schmidt , and G. Mostafaoui . 2024. “From Unimodal to Multimodal Dynamics of Verbal and Nonverbal Cues During Unstructured Conversation.” PLoS ONE 19, no. 9: e0309831. 10.1371/journal.pone.0309831. 39321138 PMC11423967

[desc70285-bib-0020] Fujiwara, K. , and I. Daibo . 2016. “Evaluating Interpersonal Synchrony: Wavelet Transform Toward an Unstructured Conversation.” Frontiers in Psychology 7: 516. 10.3389/fpsyg.2016.00516. 27148125 PMC4828427

[desc70285-bib-0021] Fujiwara, K. , M. Kimura , and I. Daibo . 2020. “Rhythmic Features of Movement Synchrony for Bonding Individuals in Dyadic Interaction.” Journal of Nonverbal Behavior 44, no. 1: 173–193. 10.1007/s10919-019-00315-0.

[desc70285-bib-0022] Fujiwara, K. , and K. Yokomitsu . 2021. “Video‐Based Tracking Approach for Nonverbal Synchrony: A Comparison of Motion Energy Analysis and OpenPose.” Behavior Research Methods 53, no. 6: 2700–2711. 10.3758/s13428-021-01612-7. 34027597

[desc70285-bib-0023] Furman, W. , and D. Buhrmester . 1992. “Age and sex Differences in Perceptions of Networks of Personal Relationships.” Child Development 63, no. 1: 103. 10.2307/1130905. 1551320

[desc70285-bib-0024] García‐Pérez, R. M. , A. Lee , and R. P. Hobson . 2007. “On Intersubjective Engagement in Autism: A Controlled Study of Nonverbal Aspects of Conversation.” Journal of Autism and Developmental Disorders 37, no. 7: 1310–1322. 10.1007/s10803-006-0276-x. 17086439

[desc70285-bib-0025] Georgescu, A. L. , S. Koeroglu , A. F. D. C. Hamilton , K. Vogeley , C. M. Falter‐Wagner , and W. Tschacher . 2020. “Reduced Nonverbal Interpersonal Synchrony in Autism Spectrum Disorder Independent of Partner Diagnosis: A Motion Energy Study.” Molecular Autism 11, no. 1: 11. 10.1186/s13229-019-0305-1. 32014017 PMC6998161

[desc70285-bib-0026] Glass, D. , and N. Yuill . 2023. “Social Motor Synchrony in Autism Spectrum Conditions: A Systematic Review.” Autism 28, no. 7: 1638–1653. 10.1177/13623613231213295. 38014541 PMC11193327

[desc70285-bib-0027] Glass, D. , and N. Yuill . 2024. “Moving Together: Social Motor Synchrony in Autistic Peer Partners Depends on Partner and Activity Type.” Journal of Autism and Developmental Disorders 54, no. 8: 2874–2890. 10.1007/s10803-023-05917-8. 37310543 PMC11300670

[desc70285-bib-0028] Hadley, L. V. , and J. A. Ward . 2021. “Synchrony as a Measure of Conversation Difficulty: Movement Coherence Increases With Background Noise Level and Complexity in Dyads and Triads.” PLoS ONE 16, no. 10: e0258247. 10.1371/journal.pone.0258247. 34610018 PMC8491905

[desc70285-bib-0029] Hale, J. , J. A. Ward , F. Buccheri , D. Oliver , and A. F. D. C. Hamilton . 2020. “Are you on my Wavelength? Interpersonal Coordination in Dyadic Conversations.” Journal of Nonverbal Behavior 44, no. 1: 63–83. 10.1007/s10919-019-00320-3. 32189820 PMC7054373

[desc70285-bib-0030] Hamilton, A. , V. Southgate , K. Miskowiak , et al. 2025. “Rhythms of Interaction—The Timescales of Social Synchrony and Why They Matter.” Neuroscience & Biobehavioral Reviews 189: 106827. https://osf.io/preprints/psyarxiv/ndjwr_v1. 10.1016/j.neubiorev.2026.106827 42398684

[desc70285-bib-0031] Hove, M. J. , and J. L. Risen . 2009. “It's All in the Timing: Interpersonal Synchrony Increases Affiliation.” Social Cognition 27, no. 6: 949–960. 10.1521/soco.2009.27.6.949.

[desc70285-bib-0032] JASP Team . 2025. JASP (Version 0.95.1) [Computer Software]. https://jasp‐stats.org/.

[desc70285-bib-0033] Kaufman, A. S. , and N. L. Kaufman . 2004. Kaufman Brief Intelligence Test–Second Edition (KBIT‐2). Pearson, Inc.

[desc70285-bib-0034] Koehler, J. C. , M. S. Dong , A. M. Bierlich , et al. 2024. “Machine Learning Classification of Autism Spectrum Disorder Based on Reciprocity in Naturalistic Social Interactions.” Translational Psychiatry 14, no. 1: 76. 10.1038/s41398-024-02802-5. 38310111 PMC10838326

[desc70285-bib-0035] Koehler, J. C. , A. L. Georgescu , J. Weiske , et al. 2022. “Brief Report: Specificity of Interpersonal Synchrony Deficits to Autism Spectrum Disorder and Its Potential for Digitally Assisted Diagnostics.” Journal of Autism and Developmental Disorders 52, no. 8: 3718–3726. 10.1007/s10803-021-05194-3. 34331629 PMC9296396

[desc70285-bib-0036] Krishnappa Babu, P. R. , J. M. Di Martino , Z. Chang , et al. 2023. “Complexity Analysis of Head Movements in Autistic Toddlers.” Journal of Child Psychology and Psychiatry 64, no. 1: 156–166. 10.1111/jcpp.13681. 35965431 PMC9771883

[desc70285-bib-0037] Kuznetsova, A. , P. B. Brockhoff , and R. H. B. Christensen . 2017. “lmerTest Package: Tests in Linear Mixed Effects Models.” Journal of Statistical Software 82, no. 13: 1–26. 10.18637/jss.v082.i13.

[desc70285-bib-0038] Ladd, G. W. 1999. “Peer Relationships and Social Competence During Early and Middle Childhood.” Annual Review of Psychology 50, no. 1: 333–359. 10.1146/annurev.psych.50.1.333. 10074682

[desc70285-bib-0039] Lord, C. , M. Rutter , P. C. DiLavore , S. Risi , K. Gotham , and S. L. Bishop . 2012. Autism Diagnostic Observation Schedule, Second Edition (ADOS‐2) Manual (part 1): Modules 124. Western Psychological Services.

[desc70285-bib-0040] Louwerse, M. M. , R. Dale , E. G. Bard , and P. Jeuniaux . 2012. “Behavior Matching in Multimodal Communication Is Synchronized.” Cognitive Science 36, no. 8: 1404–1426. 10.1111/j.1551-6709.2012.01269.x. 22984793

[desc70285-bib-0041] Martin, K. B. , Z. Hammal , G. Ren , et al. 2018. “Objective Measurement of Head Movement Differences in Children With and Without Autism Spectrum Disorder.” Molecular Autism 9, no. 1: 14. 10.1186/s13229-018-0198-4. 29492241 PMC5828311

[desc70285-bib-0042] Mayo, O. , and I. Gordon . 2020. “In and out of Synchrony—Behavioral and Physiological Dynamics of Dyadic Interpersonal Coordination.” Psychophysiology 57, no. 6: e13574. 10.1111/psyp.13574. 32221984

[desc70285-bib-0043] McDonald, D. Q. , E. Sariyanidi , C. J. Zampella , et al. 2023. “Predicting Autism From Head Movement Patterns During Naturalistic Social Interactions.” In ICMHI '23: Proceedings of the 2023 7th International Conference on Medical and Health Informatics (ICMHI). 55–60. Association for Computing Machinery. 10.1145/3608298.3608309. PMC11064057 38699395

[desc70285-bib-0044] McNaughton, K. A. , S. Dziura , E. P. Lemay , H. A. Yarger , and E. Redcay . 2025. “Neural Similarity and Interaction Success in Autistic and Non‐Autistic Adolescents.” Scientific Reports 15, no. 1: 7996. 10.1038/s41598-025-91176-9. 40055378 PMC11889181

[desc70285-bib-0045] McNaughton, K. A. , A. Moss , H. A. Yarger , and E. Redcay . 2024. “Smiling Synchronization Predicts Interaction Enjoyment in Peer Dyads of Autistic and Neurotypical Youth.” Autism 28, no. 11: 2754–2767. 10.1177/13623613241238269. PMC11408708 38497277

[desc70285-bib-0046] McNaughton, K. A. , and E. Redcay . 2020. “Interpersonal Synchrony in Autism.” Current Psychiatry Reports 22, no. 3: 12. 10.1007/s11920-020-1135-8. 32025922

[desc70285-bib-0047] Mitic, M. , K. A. Woodcock , M. Amering , et al. 2021. “Toward an Integrated Model of Supportive Peer Relationships in Early Adolescence: A Systematic Review and Exploratory Meta‐Analysis.” Frontiers in Psychology 12: 589403. 10.3389/fpsyg.2021.589403. 33716860 PMC7947339

[desc70285-bib-0048] Mogan, R. , R. Fischer , and J. A. Bulbulia . 2017. “To be in Synchrony or not? A Meta‐Analysis of Synchrony's Effects on Behavior, Perception, Cognition and Affect.” Journal of Experimental Social Psychology 72: 13–20. 10.1016/j.jesp.2017.03.009.

[desc70285-bib-0049] Noel, J.‐P. , M. A. De Niear , N. S. Lazzara , and M. T. Wallace . 2018. “Uncoupling Between Multisensory Temporal Function and Nonverbal Turn‐Taking in Autism Spectrum Disorder.” IEEE Transactions on Cognitive and Developmental Systems 10, no. 4: 973–982. 10.1109/TCDS.2017.2778141.

[desc70285-bib-0050] O'Connor, R. A. G. , N. Van Den Bedem , E. M. A. Blijd‐Hoogewys , L. Stockmann , and C. Rieffe . 2022. “Friendship Quality Among Autistic and Non‐Autistic (pre‐) Adolescents: Protective or Risk Factor for Mental Health?.” Autism 26, no. 8: 2041–2051. 10.1177/13623613211073448. 35068188 PMC9597130

[desc70285-bib-0051] Plank, I. S. , L. S. Traiger , A. M. Nelson , et al. 2023. “The Role of Interpersonal Synchrony in Forming Impressions of Autistic and Non‐Autistic Adults.” Scientific Reports 13, no. 1: 15306. 10.1038/s41598-023-42006-3. 37723177 PMC10507088

[desc70285-bib-0052] R Core Team . 2020. R: A Language and Environment for Statistical Computing [Computer software]. https://www.R‐project.org/.

[desc70285-bib-0053] Rabinowitch, T.‐C. , and A. Knafo‐Noam . 2015. “Synchronous Rhythmic Interaction Enhances Children's Perceived Similarity and Closeness Towards Each Other.” PLoS ONE 10, no. 4: e0120878. 10.1371/journal.pone.0120878. 25853859 PMC4390221

[desc70285-bib-0054] Rauchbauer, B. , and M.‐H. Grosbras . 2020. “Developmental Trajectory of Interpersonal Motor Alignment: Positive Social Effects and Link to Social Cognition.” Neuroscience & Biobehavioral Reviews 118: 411–425. 10.1016/j.neubiorev.2020.07.032. 32783968 PMC7415214

[desc70285-bib-0055] Ravreby, I. , Y. Shilat , and Y. Yeshurun . 2022. “Liking as a Balance Between Synchronization, Complexity and Novelty.” Scientific Reports 12, no. 1: 3181. 10.1038/s41598-022-06610-z. 35210459 PMC8873358

[desc70285-bib-0056] Rennung, M. , and A. S. Göritz . 2016. “Prosocial Consequences of Interpersonal Synchrony.” Zeitschrift Für Psychologie 224, no. 3: 168–189. 10.1027/2151-2604/a000252. 28105388 PMC5137339

[desc70285-bib-0057] Rifai, O. M. , S. Fletcher‐Watson , L. Jiménez‐Sánchez , and C. J. Crompton . 2022. “Investigating Markers of Rapport in Autistic and Nonautistic Interactions.” Autism in Adulthood 4, no. 1: 3–11. 10.1089/aut.2021.0017. 36600904 PMC8992924

[desc70285-bib-0058] Rubin, K. H. , W. M. Bukowski , and J. G. Parker . 2007. “Peer Interactions, Relationships, and Groups.” In Handbook of Child Psychology, edited by W. Damon and R. M. Lerner , 1st ed. Wiley. 10.1002/9780470147658.chpsy0310.

[desc70285-bib-0059] Shamay‐Tsoory, S. G. , N. Saporta , I. Z. Marton‐Alper , and H. Z. Gvirts . 2019. “Herding Brains: A Core Neural Mechanism for Social Alignment.” Trends in Cognitive Sciences 23, no. 3: 174–186. 10.1016/j.tics.2019.01.002. 30679099

[desc70285-bib-0060] Speer, S. P. H. , L. Mwilambwe‐Tshilobo , L. Tsoi , S. M. Burns , E. B. Falk , and D. I. Tamir . 2024. “Hyperscanning Shows Friends Explore and Strangers Converge in Conversation.” Nature Communications 15, no. 1: 7781. 10.1038/s41467-024-51990-7. PMC11377434 39237568

[desc70285-bib-0061] Stoit, A. M. B. , H. T. Van Schie , M. Riem , et al. 2011. “Internal Model Deficits Impair Joint Action in Children and Adolescents With Autism Spectrum Disorders.” Research in Autism Spectrum Disorders 5, no. 4: 1526–1537. 10.1016/j.rasd.2011.02.016.

[desc70285-bib-0062] Storch, E. A. , M. J. Larson , J. Ehrenreich‐May , et al. 2012. “Peer Victimization in Youth With Autism Spectrum Disorders and Co‐Occurring Anxiety: Relations With Psychopathology and Loneliness.” Journal of Developmental and Physical Disabilities 24, no. 6: 575–590. 10.1007/s10882-012-9290-4.

[desc70285-bib-0063] Tognoli, E. , M. Zhang , and J. A. S. Kelso . 2018. “On the Nature of Coordination in Nature.” In Advances in Cognitive Neurodynamics (VI), edited by J. M. Delgado‐García , X. Pan , R. Sánchez‐Campusano , and R. Wang , 375–382. Springer. 10.1007/978-981-10-8854-4_48.

[desc70285-bib-0064] Trevisan, D. A. , J. T. Enns , E. Birmingham , and G. Iarocci . 2021. “Action Coordination During a Real‐World Task: Evidence From Children With and Without Autism Spectrum Disorder.” Development and Psychopathology 33, no. 1: 65–75. 10.1017/S0954579419001561. 31896382

[desc70285-bib-0065] Tschacher, W. , G. M. Rees , and F. Ramseyer . 2014. “Nonverbal Synchrony and Affect in Dyadic Interactions.” Frontiers in Psychology 5: 1323. 10.3389/fpsyg.2014.01323. 25505435 PMC4241744

[desc70285-bib-0066] Tunçgenç, B. , and E. Cohen . 2016. “Movement Synchrony Forges Social Bonds Across Group Divides.” Frontiers in Psychology 7: 782. 10.3389/fpsyg.2016.00782. 27303341 PMC4882973

[desc70285-bib-0067] Turkstra, L. S. 2001. “Partner Effects in Adolescent Conversations.” Journal of Communication Disorders 34, no. 1–2: 151–162. 10.1016/S0021-9924(00)00046-0. 11322565

[desc70285-bib-0068] Van Den Bergh, D. , J. Van Doorn , M. Marsman , et al. 2020. “A Tutorial on Conducting and Interpreting a Bayesian ANOVA in JASP.” L'Année Psychologique 120, no. 1: 73–96. 10.3917/anpsy1.201.0073.

[desc70285-bib-0069] Van Doorn, J. , D. Van Den Bergh , U. Böhm , et al. 2021. “The JASP Guidelines for Conducting and Reporting a Bayesian Analysis.” Psychonomic Bulletin & Review 28, no. 3: 813–826. 10.3758/s13423-020-01798-5. 33037582 PMC8219590

[desc70285-bib-0070] Vicaria, I. M. , and L. Dickens . 2016. “Meta‐Analyses of the Intra‐ and Interpersonal Outcomes of Interpersonal Coordination.” Journal of Nonverbal Behavior 40, no. 4: 335–361. 10.1007/s10919-016-0238-8.

[desc70285-bib-0071] Zadok, E. , I. Gordon , R. Navon , S. J. Rabin , and O. Golan . 2022. “Shifts in Behavioral Synchrony in Response to an Interaction Partner's Distress in Adolescents With and Without ASD.” Journal of Autism and Developmental Disorders 52, no. 10: 4261–4273. 10.1007/s10803-021-05307-y. 34611838

[desc70285-bib-0072] Zhao, Z. , Z. Zhu , X. Zhang , et al. 2021. “Atypical Head Movement During Face‐to‐Face Interaction in Children With Autism Spectrum Disorder.” Autism Research 14, no. 6: 1197–1208. 10.1002/aur.2478. 33529500

