# Supplementary material for: Automated Quantification of Head Motion Synchrony in Peer Interactions of Autistic and Non‐Autistic Youth
Source: Dev Sci. 2026 Sep 14;29(6):e70285. doi: 10.1111/desc.70285 (PMC13572966; doi:10.1111/desc.70285)
Supplement: Supplementary file 1 [file DESC-29-e70285-s002.docx]

**Post-Interaction Questionnaires Supplemental Information**

*Interaction Quality (all questions on scale of 1-5 where 1=not at all, 5=a lot)*

1. How much did you enjoy the interaction?
2. How smooth/natural/relaxed was the interaction?
3. How much would you like to interact with ______ again?
4. How pleasant was the interaction?
5. How forced/strained/awkward was the interaction? (reverse scored)
6. How satisfying was the interaction?

*Desire to Interact Again – sliding scale response from -100 to 100 with 1 point increments*

If you came back for a future visit that involves interacting with another child, we could either match you with the same partner as before or a different partner. Please tell us your preference using the slider below. The more you move the slider to the left, the more strongly you feel about wanting the same partner. The more you move the slider to the right, the more strongly you feel about wanting a different partner. If you don’t feel strongly either way, move the slider to somewhere in the middle.

*Discomfort Questions*

1. I felt unsure of myself when I was with ______. (1= strongly agree, 5= strongly disagree; reverse scored)
2. How nervous did you feel when you were with ______? (1= not at all, 5= a lot)
3. How smooth/natural/relaxed was the interaction? (1=not at all, 5= a lot; reverse scored)*
4. How forced/strained/awkward was the interaction? (1=not at all, 5= a lot)*

*also included in interaction quality composite

*Supplemental Figure 1*. A multigroup confirmatory factor analysis was performed for the interaction quality measure for the participants from study 1. There was no evidence of differential item functioning between the two groups.

**Analytic Pipeline Supplemental Information**

*Supplemental Video 1*. Analysis pathway for extraction of coherence values for a brief clip from an example dyad. In the top panel, nose point coordinates for each pair of participants were extracted using OpenPose algorithms (Cao et al., 2021). Separately for each participant, frame-to-frame change in nose coordinate position was transformed into a time series of head motion, depicted in the middle panel. In the bottom panel, cross-wavelet coherence was calculated for each dyad, with warmer values indicating higher coherence and cooler values indicating lower coherence. The video provides a visual depiction of a brief example clip. For the analyses presented in the paper, further analytic steps included averaging coherence values across the time series of the interaction, resulting in 1 coherence value per dyad per frequency, as well as averaging coherence values into all six frequency bands (0.0625-0.125 Hz, 0.125-0.25 Hz, 0.25-0.5 Hz, 0.5-1 Hz, 1-2 Hz, 2-4 Hz), to aid interpretation, resulting in 1 coherence value per frequency band per dyad.

**Evidence of Significant Head Motion Synchrony in Naturalistic Peer Interactions Supplemental Information**

*Supplemental Figure 2*. True vs. pseudo synchrony in the 73 frequency values before collapsing across frequency bands. Points represent means, error bars represent ± SEM.

**Dyad Type Differences in Head Motion Synchrony Supplemental Information**

Six linear models were run, with coherence in a specific frequency band as the outcome of interest and dyad type as the predictor of interest. For the three models that were run with frequency bands that displayed evidence of above-chance coherence, the full results of those models are described in Supplemental Tables 1, 2, and 3 for frequency bands 0.0625-0.125 Hz, 0.25-0.5 Hz, and 1-2 Hz, respectively. To estimate dyad type differences within each model, dyad types were dummy-coded with rotation. Matched non-autistic dyads served as the reference group when contrasting with matched autistic and mismatched neurotype dyads. Mismatched neurotype dyads served as the reference group for contrasting matched autistic and mismatched neurotype dyads. Age, gender, data quality (proportion of frames missing from analysis/below a confidence level of 0.8), overall motion, and study (study 1 or 2) were included as covariates based on potential for confounds. Results were robust to inclusion/exclusion of covariates. The three models in which the frequency band did not have evidence of above-chance coherence are summarized for completeness of reporting in Tables 4-6, with coefficients provided for matched non-autistic vs. mismatched neurotype reported in Supplemental Table 4, coefficients for matched non-autistic vs. matched non-autistic reported in Supplemental Table 5, and coefficients for matched autistic vs. mismatched neurotype dyads in Supplemental Table 6.

*Supplemental Table 1. Lack of Dyad Type Differences in Head Motion Synchrony in the 0.0625-0.125 Hz Frequency Band*

|  | Estimate | SE | *t* | *p* |
| --- | --- | --- | --- | --- |
| Intercept | 0.295 | 0.078 | 3.763 | <0.001 |
| Dyad Type (Autistic-NonAutistic) | 0.008 | 0.025 | 0.319 | 0.751 |
| Dyad Type (Mismatched-NonAutistic) | 0.010 | 0.017 | 0.610 | 0.543 |
| Dyad Type (Autistic-Mismatched) | -0.002 | 0.025 | -0.088 | 0.930 |
| Age | -0.001 | 0.005 | -0.125 | 0.901 |
| Gender (Gender Fluid - Female) | -0.178 | 0.070 | -2.539 | 0.013 |
| Gender (Male - Female) | -0.012 | 0.016 | -0.733 | 0.466 |
| Proportion of Quality Frames | -0.234 | 0.166 | -1.408 | 0.162 |
| Motion | 0.002 | 0.001 | 1.910 | 0.059 |
| Site (Study 1 – Study 2) | -0.035 | 0.015 | -2.332 | 0.022 |

*Supplemental Table 2. Lack of Dyad Type Differences in Head Motion Synchrony in the 0.25-0.5 Hz Frequency Band*

|  | Estimate | SE | *t* | *p* |
| --- | --- | --- | --- | --- |
| Intercept | 0.294 | 0.033 | 8.956 | <0.001 |
| Dyad Type (Autistic-NonAutistic) | 0.007 | 0.010 | 0.687 | 0.494 |
| Dyad Type (Mismatched-NonAutistic) | 0.001 | 0.007 | 0.185 | 0.853 |
| Dyad Type (Autistic-Mismatched) | 0.006 | 0.011 | 0.552 | 0.582 |
| Age | 0.0002 | 0.002 | 0.100 | 0.921 |
| Gender (Gender Fluid - Female) | -0.001 | 0.029 | -0.030 | 0.976 |
| Gender (Male - Female) | -0.006 | 0.007 | -0.968 | 0.336 |
| Proportion of Quality Frames | -0.037 | 0.070 | -0.535 | 0.594 |
| Motion | 0.0004 | 0.0004 | 1.001 | 0.319 |
| Site (Study 1 – Study 2) | -0.005 | 0.006 | -0.827 | 0.410 |

*Supplemental Table 3. Lack of Dyad Type Differences in Head Motion Synchrony in the 1-2 Hz Frequency Band*

|  | Estimate | SE | *t* | *p* |
| --- | --- | --- | --- | --- |
| Intercept | 0.303 | 0.015 | 20.310 | <0.001 |
| Dyad Type (Autistic-NonAutistic) | -0.003 | 0.005 | -0.685 | 0.495 |
| Dyad Type (Mismatched-NonAutistic) | 0.002 | 0.003 | 0.636 | 0.526 |
| Dyad Type (Autistic-Mismatched) | -0.005 | 0.005 | -1.090 | 0.279 |
| Age | 0.001 | 0.001 | 1.273 | 0.206 |
| Gender (Gender Fluid - Female) | -0.012 | 0.013 | -0.906 | 0.367 |
| Gender (Male - Female) | -0.005 | 0.003 | -1.677 | 0.097 |
| Proportion of Quality Frames | -0.044 | 0.032 | -1.402 | 0.164 |
| Motion | 0.0001 | 0.0002 | -0.644 | 0.521 |
| Site (Study 1 – Study 2) | -0.004 | 0.003 | -1.504 | 0.136 |

*Supplemental Table 4. Coefficients Estimating Mismatched Neurotype vs. Matched Non-Autistic Dyad Type Differences in* *Models Run for Coherence Bands Not Displaying Evidence of Above-Chance Coherence*

| Frequency Band | Estimate | SE | *t* | *p* |
| --- | --- | --- | --- | --- |
| 0.125-0.25 Hz | -0.022 | 0.012 | -1.806 | 0.074 |
| 0.5-1 Hz | -0.004 | 0.005 | -0.811 | 0.420 |
| 2-4 Hz | -0.001 | 0.003 | -0.544 | 0.588 |

*Supplemental Table 5. Coefficients Estimating Matched Autistic vs. Matched Non-Autistic Dyad Type Differences in Models Run for Coherence Bands Not Displaying Evidence of Above-Chance Coherence*

| Frequency Band | Estimate | SE | *t* | *p* |
| --- | --- | --- | --- | --- |
| 0.125-0.25 Hz | -0.021 | 0.018 | -1.199 | 0.234 |
| 0.5-1 Hz | 0.002 | 0.007 | 0.332 | 0.741 |
| 2-4 Hz | -0.004 | 0.004 | -0.989 | 0.325 |

*Supplemental Table 6. Coefficients Estimating Matched Autistic vs. Mismatched Dyad Type Differences in Models Run for Coherence Bands Not Displaying Evidence of Above-Chance Coherence*

| Frequency Band | Estimate | SE | *t* | *p* |
| --- | --- | --- | --- | --- |
| 0.125-0.25 Hz | 0.0002 | 0.018 | 0.011 | 0.992 |
| 0.5-1 Hz | 0.006 | 0.007 | 0.859 | 0.393 |
| 2-4 Hz | -0.003 | 0.004 | -0.613 | 0.542 |

Follow-up Bayesian ANOVAs were run in JASP v0.95.1 to quantify evidence for and against the effect of dyad type (matched autistic, matched non-autistic, and mismatched neurotype) explaining variance in synchrony. Six Bayesian ANOVAs were run with uniform priors, with coherence in each of the six frequency band as the outcome and dyad type as a between-subjects factor. The Bayes factors for including and excluding the effect of dyad type in explaining the variance in synchrony in each frequency band are summarized in Supplemental Table 7.

*Supplemental Table 7. Results of Bayesian ANOVAs for Effect of Dyad Type in Each Frequency Band*

| Frequency Band | BF_includeDyadType_ | BF_excludeDyadType_ |
| --- | --- | --- |
| 0.0625-0.125 Hz | 0.12 | 8.352 |
| 0.125-0.25 Hz | 1.606 | 0.623 |
| 0.25-0.5 Hz | 0.122 | 8.198 |
| 0.5-1 Hz | 0.145 | 6.906 |
| 1-2 Hz | 0.305 | 3.281 |
| 2-4 Hz | 0.237 | 4.222 |

**Relations Between Head Motion Synchrony and Interaction Enjoyment**

**Supplemental Information**

To assess relations between synchrony and interaction outcomes (interaction quality, desire to interact again, discomfort), multilevel models were constructed treating youth-reported interaction enjoyment as nested within dyads, and intercepts were modeled as randomly varying across dyads. The discomfort outcome was square-root transformed to better meet normality assumptions. Coherence in a given frequency band was the predictor of interest, and time-scrambled pseudocoherence, age, gender, dyad type (matched autistic/mismatched neurotype/matched non-autistic), data quality (proportion of frames missing from analysis/below a confidence level of 0.8), overall motion, and study (study 1 or 2, for the interaction quality outcome that was collected across both studies) were included as covariates of no interest. Results were robust to inclusion/exclusion of covariates.

Supplemental Table 8 summarizes the results of a multilevel model predicting interaction quality from head motion synchrony in the 0.25-0.5 Hz coherence band.

Supplemental Table 9 summarizes the results of a multilevel model predicting desire to interact again from head motion synchrony in the 0.25-0.5 Hz coherence band.

Supplemental Table 10 summarizes the results of a multilevel model predicting discomfort from head motion synchrony in the 0.25-0.5 Hz coherence band.

Supplemental Tables 11-13 summarize the results of 15 multilevel models, providing only the coefficient for the effect of synchrony in a specified coherence band on an interaction outcome. These models either examined relations between synchrony and interaction enjoyment that did not reach significance, or examined relations between synchrony and interaction enjoyment in coherence bands that did not display evidence of above-chance coherence. These results are provided for completeness of reporting.

*Supplemental Table 8. Predicting Interaction Quality from Head Motion Synchrony in the 0.25-0.5 Hz Frequency Band*

|  | Estimate | SE | df | *t* | *p* |
| --- | --- | --- | --- | --- | --- |
| Intercept | 32.13 | 7.25 | 90.40 | 4.43 | <0.001 |
| Synchrony | -21.29 | 14.63 | 88.07 | -1.46 | 0.15 |
| Time-Scrambled Pseudosynchrony | -7.53 | 16.14 | 88.07 | -0.47 | 0.64 |
| Age | -0.38 | 0.26 | 98.00 | -1.50 | 0.14 |
| Gender (Gender Fluid - Female) | 2.43 | 4.04 | 88.10 | 0.60 | 0.55 |
| Gender (Male - Female) | -1.20 | 0.94 | 88.46 | -1.29 | 0.20 |
| Dyad Type (Autistic-NonAutistic) | 2.57 | 1.43 | 88.09 | 1.80 | 0.08 |
| Dyad Type (Mismatched-NonAutistic) | 0.39 | 0.95 | 88.56 | 0.41 | 0.68 |
| Proportion of Quality Frames | -17.15 | 10.06 | 88.11 | -1.70 | 0.09 |
| Overall Motion | 0.13 | 0.07 | 88.20 | 1.92 | 0.06 |
| Site (Study 1 – Study 2) | 0.24 | 0.90 | 88.15 | 0.27 | 0.79 |

*Supplemental Table 9. Predicting Desire to Interact Again from Head Motion Synchrony in the 0.25-0.5 Hz Frequency Band*

|  | Estimate | SE | df | *t* | *p* |
| --- | --- | --- | --- | --- | --- |
| Intercept | 177.05 | 96.32 | 41.89 | 1.838 | 0.07 |
| Synchrony | -583.07 | 200.47 | 40.57 | -2.91 | 0.01 |
| Time-Scrambled Pseudosynchrony | -41.86 | 252.21 | 41.22 | -0.17 | 0.87 |
| Age | 1.91 | 3.55 | 45.97 | 0.54 | 0.59 |
| Gender (Male-Female) | -22.06 | 13.58 | 41.75 | -1.63 | 0.11 |
| Dyad Type (Autistic-NonAutistic) | 29.25 | 23.39 | 40.62 | 1.25 | 0.22 |
| Dyad Type (Mismatched-NonAutistic) | -43.11 | 16.61 | 45.65 | -2.60 | 0.01 |
| Proportion of Quality Frames | -84.17 | 192.88 | 41.04 | -0.44 | 0.66 |
| Overall Motion | 0.91 | 0.91 | 43.82 | 1.00 | 0.32 |

*Supplemental Table 10. Predicting Youth-Reported Discomfort from Head Motion Synchrony in the 0.25-0.5 Hz Frequency Band*

|  | Estimate | SE | df | *t* | *p* |
| --- | --- | --- | --- | --- | --- |
| Intercept | 0.54 | 1.01 | 49.76 | 0.54 | 0.60 |
| Synchrony | 5.58 | 2.05 | 49.07 | 2.72 | 0.01 |
| Time-Scrambled Pseudosynchrony | 2.53 | 2.57 | 49.07 | 0.98 | 0.33 |
| Age | 0.04 | 0.03 | 52.87 | 1.19 | 0.24 |
| Gender (Male-Female) | 0.13 | 0.14 | 49.33 | 0.96 | 0.34 |
| Dyad Type (Autistic-NonAutistic) | -0.46 | 0.23 | 49.07 | -2.00 | 0.05 |
| Dyad Type (Mismatched-NonAutistic) | -0.01 | 0.15 | 49.45 | -0.10 | 0.92 |
| Proportion of Quality Frames | 2.75 | 1.81 | 49.09 | 1.52 | 0.14 |
| Overall Motion | -0.02 | 0.01 | 49.10 | -1.69 | 0.10 |

There were no significant relations between synchrony and interaction quality (Supplemental Table 11), synchrony and desire to interact again (Supplemental Table 12), and synchrony and discomfort (Supplemental Table 13) in the 0.0625-0.125 Hz, 0.5-1 Hz, 1-2 Hz, and 2-4 Hz frequency bands. There were significant relations between synchrony and interaction quality, desire to interact again, and discomfort in the 0.125-0.25 Hz frequency band.

*Supplemental Table 11. Coefficients for Relations Between Interaction Quality and Synchrony in the 0.0625-0.125 Hz, 0.125-0.25 Hz, 0.5-1 Hz, 1-2 Hz, and 2-4 Hz Frequency Bands*

|  | Estimate | SE | df | *t* | *p* |
| --- | --- | --- | --- | --- | --- |
| 0.0625-0.125 Hz Synchrony | 4.43 | 6.40 | 88.08 | 0.69 | 0.49 |
| 0.125-0.25 Hz Synchrony | -20.42 | 8.28 | 88.07 | -2.47 | 0.02 |
| 0.5-1 Hz Synchrony | 3.92 | 22.32 | 88.08 | 0.18 | 0.86 |
| 1-2 Hz Synchrony | -28.41 | 32.41 | 88.25 | -0.88 | 0.38 |
| 2-4 Hz Synchrony | -12.10 | 41.45 | 88.08 | -0.29 | 0.77 |

*Supplemental Table 12. Coefficients for Relations Between Desire to Interact Again and Synchrony in the 0.0625-0.125 Hz, 0.125-0.25 Hz, 0.5-1 Hz, 1-2 Hz, and 2-4 Hz Frequency Bands*

|  | Estimate | SE | df | *t* | *p* |
| --- | --- | --- | --- | --- | --- |
| 0.0625-0.125 Hz Synchrony | 47.55 | 104.29 | 41.08 | 0.46 | 0.65 |
| 0.125-0.25 Hz Synchrony | -260.23 | 125.21 | 40.78 | -2.08 | 0.04 |
| 0.5-1 Hz Synchrony | -456.46 | 382.72 | 41.37 | -1.19 | 0.24 |
| 1-2 Hz Synchrony | 530.88 | 551.60 | 40.94 | 0.96 | 0.34 |
| 2-4 Hz Synchrony | 98.79 | 680.22 | 41.16 | 0.15 | 0.89 |

*Supplemental Table 13. Coefficients for Relations Between Youth-Reported Discomfort and Synchrony in the 0.0625-0.125 Hz, 0.125-0.25 Hz, 0.5-1 Hz, 1-2 Hz, and 2-4 Hz Frequency Bands*

|  | Estimate | SE | df | *t* | *p* |
| --- | --- | --- | --- | --- | --- |
| 0.0625-0.125 Hz Synchrony | -0.66 | 1.01 | 49.12 | -0.65 | 0.52 |
| 0.125-0.25 Hz Synchrony | 2.95 | 1.32 | 49.08 | 2.24 | 0.03 |
| 0.5-1 Hz Synchrony | -1.36 | 3.70 | 49.08 | -0.37 | 0.72 |
| 1-2 Hz Synchrony | -4.69 | 5.66 | 49.28 | -0.83 | 0.41 |
| 2-4 Hz Synchrony | 7.76 | 6.83 | 49.25 | 1.14 | 0.26 |

*Supplemental Figure 3*. Relations between synchrony and interaction outcomes visualized by dyad type. There were no significant statistical interactions between dyad type and head motion in predicting interaction outcomes that remained after correction for multiple comparisons across frequency bands (*p*s>0.05).

**Residual Plots**

*Supplemental Figure 4.* Residual plots for models visualized in Figure 3.

*Supplemental Figure 5.* Residual plots for models visualized in Figure 4.
